# The Chinese Herbal Formula Huoxiang Zhengqi Dropping Pills Prevents Acute Intestinal Injury Induced by Heatstroke by Increasing the Expression of Claudin-3 in Rats

**DOI:** 10.1155/2022/9230341

**Published:** 2022-07-31

**Authors:** Yu-Nong Li, Hui Tao, Jia-Hui Hong, Ya-Lan Xiong, Xi-Chun Pan, Ya Liu, Xue-Sen Yang, Hai-Gang Zhang

**Affiliations:** ^1^Department of Pharmacology, College of Pharmacy, Army Medical University (Third Military Medical University), Chongqing, China; ^2^Department of Tropical Medicine, College of Military Preventive Medicine, Army Medical University (Third Military Medical University), Chongqing, China

## Abstract

Intestinal injury has been regarded as an important causative factor for systemic inflammation during heatstroke, and maintaining intestinal integrity has been a potential target for the prevention of HS. Huoxiang Zhengqi Dropping Pills (HZPD) is a modern preparation of Huoxiang Zhengqi and widely used to prevent HS. The present study aims to explore the protective effect of HZDP on intestinal injury during heatstroke and analyze its potential pharmacodynamic basis. Male rats in the control and HS groups were given normal saline, and those in the HZDP groups were given HZDP (0.23, 0.46, and 0.92 g/kg) before induction of HS. Serum contents of tumor necrosis factor-*α* (TNF-*α*), interleukin-6 (IL-6), intestinal fatty acid-binding protein (iFABP), and diamine oxidase (DAO) were determined using ELISA. Histopathology of intestinal injury was observed following H&E staining. The expression of claudin-3 was determined using western blot, immunohistochemistry, and immunofluorescence techniques. Moreover, network pharmacological tools were used to analyze the potential pharmacodynamic basis and the mechanism of HZDP. Treatment with HZDP significantly prolonged the time to reach Tc. Compared with the control group, the contents of TNF-*α*, IL-6, iFABP, and DAO in HS rats increased markedly. HZDP treatments reduced these levels significantly, and the effects in the middle dose group (0.46 g/kg) were most obvious. HZDP also attenuated intestinal injury and significantly reversed the decrease in claudin-3 expression. Bioinformatics analysis suggested that 35 active ingredients and 128 target genes of HZDP were screened from TCMSP and 93 target genes intersected with heatstroke target genes, which were considered potential therapeutic targets. TNF-*α* and IL-6 were the main inflammatory target genes of HZDP correlated with HS. These results indicated that HZDP effectively protected intestinal barrier function and prevented acute intestinal injury by increasing the expression of claudin-3 in rats, eventually improving heat resistance.

## 1. Introduction

Continuous global warming results in a rapid increase in the mortality risk of heat-related illnesses, which seriously restricts the efficiency of labor productivity for outdoor work. Heatstroke (HS) is the most serious type of heat injury disease and is characterized by elevated core temperatures (Tc) as high as 40°C, systemic inflammatory response syndrome (SIRS), and multiple organ dysfunction (MOD) [1]. Although rapid cooling methods and a few drugs for HS therapy have been applied, SIRS and MOD still occur frequently [1, 2]. Because of the rapid progression, high mortality, and limitations of current clinical drugs in the treatment of HS, preventative strategies are more clinically effective than treatment after onset.

Heat exposure induces tissue damage, including hepatic, renal, and intestinal injuries in humans, and intestinal injury is the main route of HS progression [3, 4]. Heat stress impairs intestinal barrier integrity and intestinal permeability, causing endotoxin translocation into systemic circulation, which plays a key role in aggravating systemic inflammation [5–7]. Therefore, preventing injury to the intestinal mucosal barrier may be an effective modality for patients suffering from HS [8, 9].

The main manifestation of intestinal injury is the impairment of barrier function. Intestinal barrier function is achieved by intercellular junctions, including subjacent adherens junctions (AJs) and apical tight junctions (TJs). The TJs between intestinal epithelial cells present a robust barrier to invasion by bacteria and their toxins. Defective TJs resulting in intestinal barrier dysfunction are the primary reason for HS-related alterations in intestinal permeability [8, 10]. The TJs consist of transmembrane proteins (occludin, claudins, and junction adhesion molecules), intracellular plaque zonula occludens (ZO-1, ZO-2, and ZO-3), and other related kinases [8, 11, 12]. The claudin family of transmembrane proteins plays a critical role in maintaining tight junction integrity and may regulate the selective passage of ions and molecules through the paracellular space [13]. Abnormal expression of claudins causes changes in paracellular permeability, abnormal cell proliferation, loss of polarity, and obstacles to differentiation and participates in tumorigenesis [14].

Claudin-3 is one of the 26 claudin family members, and its effect on intestinal function has been reported. Increasing expression of claudin-3 can strengthen the connection between cells and reduce paracellular permeability [15]. Another study also reported that hyperthermia exposure disrupted the established monolayer by increasing paracellular permeability and decreasing claudin-3 expression at 42°C [16]. However, the altering pattern of the expression and distribution of claudin-3 and its regulation of TJs and intestinal barrier function during heat exposure remain unclear.

Huoxiang Zhengqi Dropping Pills (HZDP) is a Chinese patent medicine composed of 10 kinds of traditional Chinese herbs, including Atractylodis Rhizoma, Citri Reticulatae Pericarpium, Magnoliae Officinalis Cortex, Atractylodis Macrocephalae Rhizoma, Poria, Arecae Pericarpium, Pinelliae Rhizoma, Glycyrrhizae Radix Et Rhizoma, Pogostemonis Herba, and Perillae Folium [17, 18]. It was modified in dosage form based on a traditional formula named Huoxiang Zhengqi powder, which originated from the ancient Chinese pharmacy book *Taiping Huimin Hejiju Fang* and has been traditionally used to treat acute gastroenteritis and gastrointestinal injury associated with the dampness pattern in Chinese medicine [19–22]. A variety of studies have shown that Huoxiang Zhengqi powder could treat acute heatstroke [23, 24] and alleviate dampness pattern in rat models through normalizing gastrointestinal dysfunction [25] and anti-inflammatory and intestinal function-modulating activities [26]. Li have ever treated 240 cases of infantile high fever effectively using Huoxiang Zhengqi water [27]. Additionally, it was also reported that Pogostemonis Herba, a main component of HZDP, could alleviate heat stress [28]. Magnolol, the main active substance of Magnoliae Officinalis Cortex, could protect against cerebral ischemic injury of rat heatstroke [29]. However, few studies have focused on the mechanism of HZDP in the treatment of HS. In the present study, experiments were designed to confirm the protective effect of HZDP on acute intestinal injury induced by HS and to explore potential mechanisms in a humidified/heat environment. Moreover, the potential pharmacodynamic basis and molecular mechanism of HZDP in acute intestinal injury were analyzed by network pharmacological tools.

## 2. Materials and Methods

### 2.1. Animals

Male Sprague–Dawley (SD) rats weighing 130–150 g were purchased from the Animal Center of the Army Medical University (Chongqing, China). Animals were housed in a room maintained at 25.0 ± 0.5°C and humidity of 60 ± 5% and were given free access to standard laboratory chow and water. Male rats were randomly assigned to the control, HS, and HZDP-treated (0.23, 0.46, and 0.92 g/kg) groups. Rats in the control and HS groups were intragastrically (ig) administered normal saline, and those in the HZDP groups were given HZDP solution at 0.23, 0.46, and 0.92 g/kg (ig) once daily for 7 days before induction of HS. All animal procedures conformed to the Guide for the Care and Use of Laboratory Animals from the National Institutes of Health (8th Ed., 2011) and were approved by the Animal Ethical and Experimental Committee of the Army Medical University.

### 2.2. Quality Control of HZDP

The component content of Huoxiang Zhengqi Dropping Pills (Tasly Pharmaceutical Group, Lot number 200707, Tianjin, China) was determined by a high-performance liquid chromatography (HPLC) system (Agilent Infinity 1260; Agilent Technologies, Santa Clara, CA, USA). The chromatographic column was filled with octadecylsilane bonded silica gel, acetonitrile as mobile phase A, and 0.5% glacial acetic acid solution as mobile phase B, and the theoretical plate number calculated by hesperidin was not less than 2500. For preparation of the sample solution, 1 g of HZDP was crushed, weighed accurately, and placed in a conical flask with a stopper. In addition, then 25 mL of methanol was added into the flask. The samples were treated with ultrasound (120 W, 40 kHz) for 15 minutes, weighed after cooling, supplemented the lost weight with methanol, shaken, and filtered to obtain the filtrate. For preparation of the reference solution, the reference standards of hesperidin, magnolol, and honokiol (Chengdu Must Bio-Technology, Chengdu, China) were weighed accurately, and methanol was added to prepare the reference solution. The final concentrations of hesperidin, magnolol, and honokiol were 140 *μ*g/mL, 40 *μ*g/mL, and 70 *μ*g/mL, respectively. One microliter of each solution was injected. The sample solution was analyzed, and the peaks were identified compared with the available standards.

### 2.3. HS Injury Rat Model Induction

Twenty-five rats were randomly assigned to five groups (*n* = 5 in each group), i.e., control, HS, and HZDP-treated (0.23, 0.46, and 0.92 g/kg) groups. Rats in the control group and HS group were intragastrically (ig) administered normal saline, and those in the HZDP groups were given HZDP solution at 0.23, 0.46, and 0.92 g/kg (ig) once daily for 7 days before being exposed to a humid-heat environment. The animals in the control group were sham-heated at a temperature of 25 ± 0.5°C and humidity of 60 ± 5%. Rats in the HS group and HZDP-treated HS groups were placed in a specific environment-control smart chamber (HOPE-MED 8150E; Tianjin, China) at 38°C and 90% humidity. Core temperature (Tc) was monitored every 10 minutes using a rectal thermometer until it reached 42°C, which indicated the occurrence of heat radiation disease.

### 2.4. Serum Collection and Determination of Inflammatory Factors and Markers of Intestinal Integrity

HS rats were anesthetized by isoflurane 2 h after the onset of HS. Blood samples were harvested from the femoral artery, and serum was separated by centrifugation at 3,000 rpm for 10 min at 4°C and stored at −80°C for ELISA analysis. ELISA kits were used to measure the serum levels of TNF-*α* and IL-6 (Invitrogen, CA, USA) and iFABP and DAO (Elabscience, Wuhan, China) according to the manufacturer's instructions.

### 2.5. Histopathological Examination

Samples of jejunum were harvested at 6 h after HS, sliced into transverse sections, and fixed in 10% neutral-buffered formalin. The tissues were then embedded in paraffin blocks after conventional gradient ethanol dehydration, sectioned into 5 *µ*m-thick slices, stained with hematoxylin and eosin (H&E), and examined microscopically at a magnification of 200×.

### 2.6. Immunohistochemistry Staining

Paraffin-embedded jejunum sections were used for immunohistochemistry staining for claudin-3. Samples were dewaxed and hydrated through graded ethanol solutions, repaired antigen with citrate buffer (pH 6.0), blocked endogenous peroxidase with 3% H2O2 for 20 minutes at room temperature, blocked in goat serum, and then incubated with primary antibody claudin-3 (1:200, Invitrogen, CA, USA) overnight at 4 °C. After incubation with the appropriate biotin-labeled secondary antibody and subsequently with HRP-labeled streptavidin, color reaction was developed with diaminobenzidine. And next steps were counterstained with hematoxylin, differentiation with 1% hydrochloric acid-alcohol solution, dehydration, transparency, and sealing.

### 2.7. Immunofluorescence Staining

Paraffin-embedded sections of the jejunum samples were used for claudin-3 immunofluorescence staining. Claudin-3 was stained with claudin-3 primary antibody (1 : 50) and Alexa Fluor 594-conjugated secondary antibody (1 : 300, Invitrogen, CA, USA), and the nuclei were stained with DAPI. The specimens were observed and imaged using laser confocal microscopy (TCS SP8; Leica, Biberach, Germany).

### 2.8. Western Blot Analysis

Samples of jejunum tissue were harvested at 6 h after HS and stored at −80°C. Tissue was suspended in RIPA lysis buffer (Beyotime Biotechnology, Shanghai, China) supplemented with 10 mM PMSF and then ground using an automatic sample freezing grinding instrument at 4°C. Tissue lysates were harvested by centrifugation at 12000 *g* for 15 minutes at 4°C. Protein concentrations were measured using the enhanced BCA protein assay kit (Beyotime Biotechnology, Shanghai, China). Lysates were subjected to SDS/PAGE followed by blotting with primary antibody against claudin-3 (1 : 1000) and corresponding secondary antibody (1 : 5000, Minneapolis, USA). Signal detection was achieved using Clarity Western ECL substrate (Bio-Rad, CA, USA), and the intensity was captured using a ChemiDoc touch system (Bio-Rad, CA, USA).

### 2.9. Network Pharmacological Analysis

To understand the potential target and mechanism of HZDP, we performed network pharmacological analysis with bioinformatics tools. The ingredients of HZDP were searched from the Traditional Chinese Medicine Systems Pharmacology Database and Analysis Platform (TCMSP, https://tcmspw.com/index.php) based on oral bioavailability (OB) ≥ 30% and drug-likeness (DL) ≥ 0.18. The active ingredient-related target genes were obtained from the TCMSP, and these target names were calibrated to the standardized name in the UniProt database (https://www.UniProt.org/). HS-related target genes were collected from GeneCard (https://www.genecards.org/) and Online Mendelian Inheritance in Man (OMIM, https://omim.org/search/advanced/geneMap). Potential target genes of HZDP that prevented HS were acquired through Venny 2.1.0 (https://bioinfogp.cnb.csic.es/tools/venny/). A PPI network map of potential targets was acquired through the STRING database (https://string-db.org/). The PPI network results were imported into Cytoscape software (version 3.9.0), and the network topology parameters were analyzed by the degree calculated to select the key target genes.

### 2.10. Statistical Analysis

Results are expressed as the mean ± SD. Statistical comparisons of the results were performed using one-way analysis of variance (ANOVA) following least significant difference (LSD) post hoc analyses using SPSS software 11.0 (Chicago, IL, USA). Time to core temperature between HS and HZDP-treated groups were analyzed with Dunnett's method. *P* < 0.05 was considered statistically significant.

## 3. Results

### 3.1. Active Ingredient Content of HZDP

**Figure 1:**
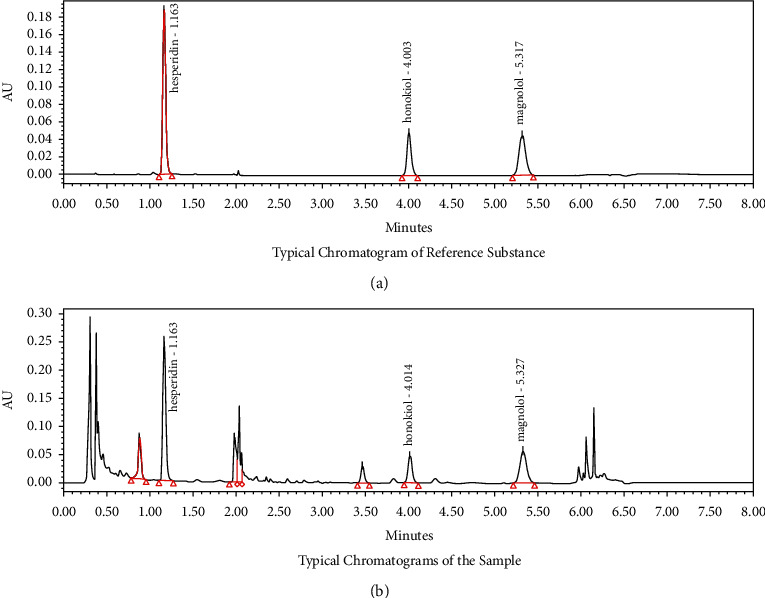
HPLC analysis of Huoxiang Zhengqi dropping pills (HZDP) component content. (a) HPLC chromatogram of reference substance and (b) HPLC chromatogram of sample substance.

HZDP is a compound preparation composed of a variety of traditional Chinese medicines. The contents of hesperidin, magnolol, and honokiol were used as HZDP quality control standards. As shown in Figure 1(a), the retention times of standards of hesperidin, magnolol, and honokiol in the reference substance were 1.163, 4.003, and 5.317 minutes, respectively. In the chromatogram of the sample substance (Figure 1(b)), we found three typical chromatographic peaks, and their retention times were the same as those of the standards. In the HZDP sample used in the present experiments, the contents of hesperidin, magnolol, and honokiol were 4.33 mg/g, 0.94 mg/g, and 2.07 mg/g, respectively.

### 3.2. HZDP Improves High-Temperature Resistance during Heat Exposure

**Figure 2:**
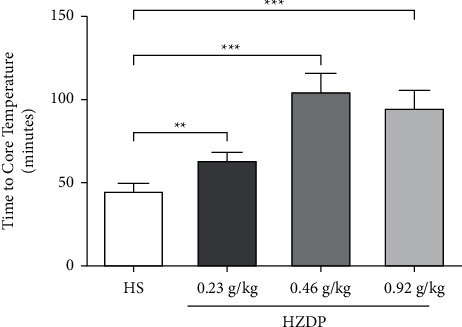
Huoxiang Zhengqi dropping pills (HZDP) prolonged the time to core temperature (Tc) of 42°C in rats. Rats were exposed to 38°C, 90% relative humidity. Data were presented as mean ± SD (n = 5), ^∗∗^*P* < 0.01, ^∗∗∗^*P* < 0.001 compared with HS group.

To verify the ability of HZDP to prevent HS, rats were pretreated with HZDP prior to the establishment of the HS model. Under an environment of high temperature and humidity, rats gradually became irritable, hyperactive, and hyperhidrotic and finally fell into less activity and coma. When the core temperature (Tc) of rats reached 42°C, heat radiation disease had occurred. The time to reach Tc was used to evaluate the effects of HZDP on high-temperature resistance. Compared with the HS group, the rats in HZDP-treated groups were quieter and sweated less, and the time to reach Tc elevated significantly. In particular, the time to reach Tc of rats in the 0.46 g/kg HZDP-treated HS group was longer than that of the other two groups (0.23 and 0.92 g/kg) (Figure 2). This result suggests that pretreatment with HZDP can improve the high-temperature and high-humidity resistance.

### 3.3. HZDP Ameliorates Intestinal Pathologic Injury Induced by HS

**Figure 3:**
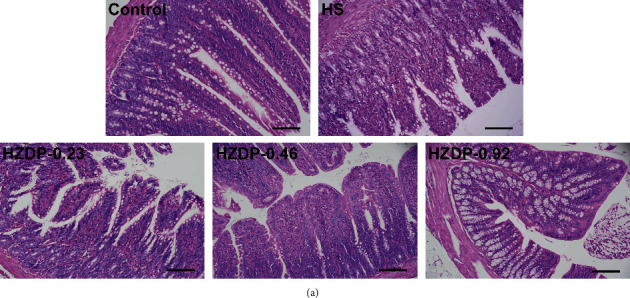
Huoxiang Zhengqi dropping pills (HZDP) preadministration attenuated intestinal injury in rats during heatstroke. Representative pathological images of jejunum from control rats, HS rats, and HZDP-treated HS rats stained with H&E. In each group, n = 5. Scale bar =50 μm.

To further explore the effect of HZDP on intestinal injury, histopathological examination was performed on intestinal tissue. In the HS group, the injury of rat jejunum mainly located in the villi with loss of the integrity of the epithelial lining, interstitial edema in the intestinal villi, apparent shortening, and villus desquamating. In contrast, in the HZDP-treated HS groups, although morphology changed to a certain degree, the villi of the jejunum were relatively protected from injury, as villus swelling and desquamating decreased. Among those, 0.46 g/kg HZDP treatment had the best effect on intestinal protection (Figure 3).

### 3.4. HZDP Improves Intestinal Mucosa Barrier Function during Heat Exposure

**Figure 4:**
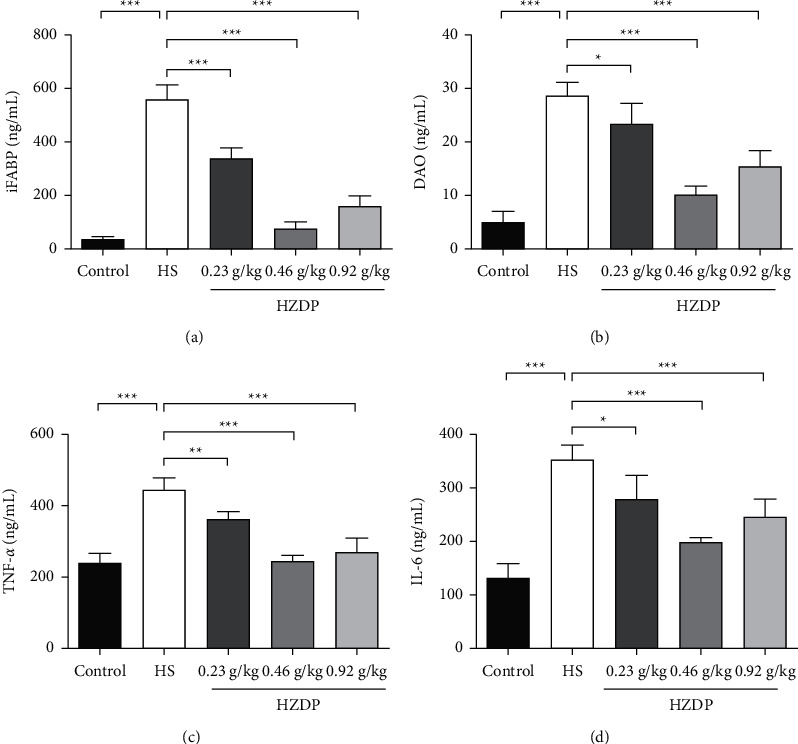
Huoxiang Zhengqi dropping pills (HZDP) preadministration decreased levels of intestinal-related proteins and inflammatory cytokines. Serum concentrations of intestinal fatty acid binding protein (iFABP) (a), diamine oxidase (DAO) (b), tumor necrosis factor-α (TNF-α) (c), and interleukin- 6 (IL-6) (d), were presented as mean ± SEM (n = 5), ^∗^*P* < 0.05, ^∗∗^*P* < 0.01, ^∗∗∗^*P* < 0.001 compared with HS group.

When the intestinal mucosa is damaged, the serum concentrations of iFABP and DAO will increase, which are normally absent from systemic circulation. Compared with the control group, the levels of serum iFABP and DAO in the HS group were significantly elevated. Pretreatment with HZDP significantly reduced the levels compared with that of the HS group (Figures 4(a) and 4(b)). In addition, the serum inflammatory cytokines TNF-*α* and IL-6 were selected to evaluate the inflammatory response. The levels of serum TNF-*α* and IL-6 increased during heat exposure, but those in the HZDP-treated HS groups were lower than those in the HS group (Figures 4(c) and 4(d)). These results suggest that HZDP improves the intestinal mucosa barrier and reduces intestinal permeability during heat exposure, further reducing bacterial and endotoxin translocation and resulting in a systemic inflammatory response.

### 3.5. HZDP Reverses the Decrease in Claudin-3 Expression

**Figure 5:**
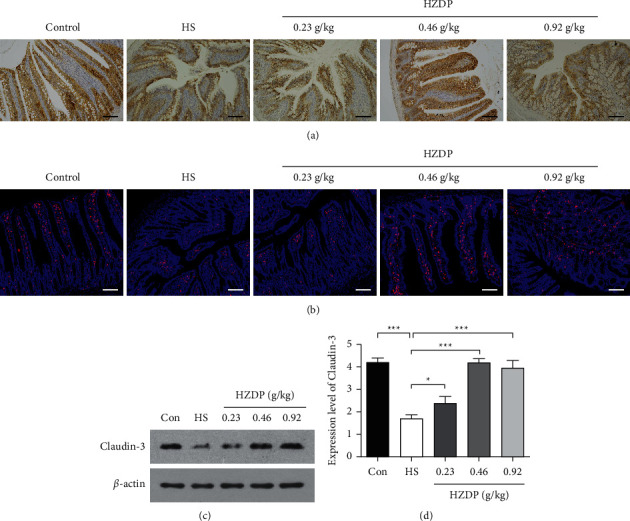
HZDP reversed the decrease of claudin-3 caused by heat stress. Immunohistochemistry (a) and immunofluorescence (b) analysis showed the expression of claudin-3. In each group, n = 5. Scale bar = 50 μm. The expression of claudin-3 was detected by western blot analysis (c and d). Data were presented as mean ± SD (n = 5, ^∗^*P* < 0.05, ^∗∗∗^P < 0.001).

Since claudin-3 is the main component of TJs, we explored the effect of HZDP on claudin-3 expression during heat exposure. Compared with the control group, the expression of claudin-3 decreased remarkably in the HS group. Simultaneously, HZDP pretreatment reversed this reduction. We found that the expression of claudin-3 increased in the HZDP-treated HS groups (Figures 5(a) and 5(b)), and the level of claudin-3 in the 0.46 g/kg HZDP group recovered to a similar level as that in the control group (Figures 5(c) and 5(d)).

### 3.6. Potential Target Genes and the PPI Network Map of HZDP Treatment for Heatstroke

**Figure 6:**
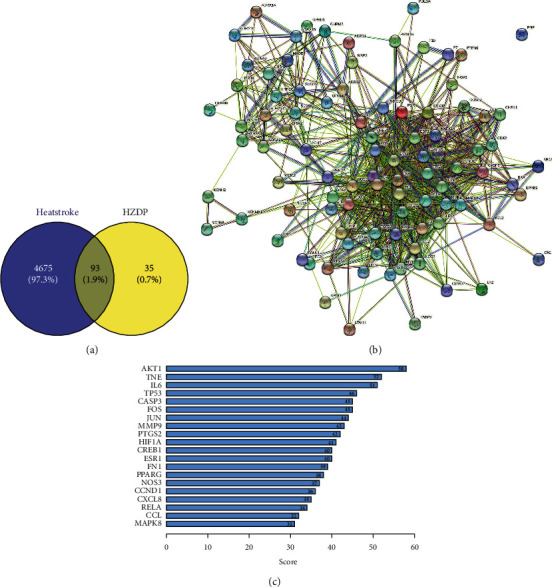
Potential target genes and PPI network map of Huoxiang Zhengqi dropping pills (HZDP) treatment for heatstroke. (a) The potential target genes of HZDP treatment for HS in Venny. (b) The PPI network map of 96 target genes. (c) The list of top 20 genes of PPI network map.

HZDP ingredients were searched from the TCMSP database, and 35 active ingredients were selected (Supplementary Table S1). A total of 4768 HS target genes were obtained from the GeneCard and OMIM databases. One hundred and twenty-eight target genes, excluding duplicates, were obtained from the TCMSP corresponding to 35 active ingredients of HZDP (Supplementary Table S2). HS-related genes and HZDP target genes were intersected using Venny 2.1.0 to obtain 93 potential targets (Figure 6(a) and Table 1). Furthermore, the 93 potential targets were imported into the STRING database to obtain the PPI network map (Figure 6(b)). The results of PPI network were analyzed by the degree calculated in Cytoscape software, and the top 20 key target genes were acquired (Figure 6(c)). Inflammatory target genes such as TNF and IL-6 were the main key target genes. Therefore, we speculate that inflammation plays an important role in the pathophysiological process of heatstroke and the effect of HZDP is closely related to it.

## 4. Discussion

Accumulating evidence has indicated that the main mortality from heatstroke (HS) is the result of systemic inflammatory response syndrome (SIRS). Bacterial translocation and endotoxemia induced by intestinal injury and increased permeability are related to the pathophysiological process of SIRS [30, 31]. Preventing and mitigating intestinal injury could be considered a potential clinical strategy to minimize the incidence of HS [8].

We found different protective effects on rats treated with different doses of HZDP, among which 0.46 g/kg HZDP treatment had the best effect on intestinal protection. Additionally, the time to Tc in this group was longest compared with the other two groups. This might be because 0.46 g/kg HZDP was the maximum therapeutic dose, while 0.92 g/kg HZDP caused some side effects. We hypothesized that raw Pinelliae Rhizoma, one of the components of HZDP, was the main cause of side effects. Studies have shown that raw Pinelliae Rhizoma has a stimulating effect on gastrointestinal mucosa and could lead to gastrointestinal mucosal damage, even inflammation, intestinal ulcers, and bleeding overdose [32, 33]. Zhu et al. and Yu et al. [34, 35] have reported that calcium oxalate needle crystals and Pinellia lectin proteins were the main toxic components of raw Pinelliae Rhizoma, and calcium oxalate needle crystals were the main components of raw Pinellia Rhizoma to produce irritant adverse reactions. Calcium oxalate needle crystals had long and thin tip, barb, and trough and caused irritation by piercing into the tissue through pressure and the force of mucus cells. Moreover, Pinellia lectin proteins, attaching to calcium oxalate needle crystals, then entered the tissue and caused inflammation. Experiments confirmed that intragastric administration of tubers of raw Pinelliae Rhizoma at a dose of 0.5 g/kg for 3 days to rats significantly decreased the content of prostaglandin E_2_ (PGE_2_), inducing serious damage of gastric mucosa [36]. High dose of HZDP (0.92 g/kg) may also cause gastrointestinal mucosal damage by reducing PGE_2_, but whether the specific components producing toxicity are only calcium oxalate needle crystals and Pinellia lectin proteins and the mechanism of toxicity need to be further confirmed. Interestingly, this optimal dose (0.46 g/kg) in rats is equivalent to the daily clinical dosage for adult humans (5.2 g).

Heat stress could impair intestinal barrier integrity by increasing intestinal permeability, resulting in the increase of plasma endotoxin in the circulation and leading to severe conditions [37, 38]. Molecules related to this process might be used as indicators for evaluating the integrity and permeability of the intestinal barrier. Intestinal fatty acid-binding protein (iFABP), located at the luminal pole of the enterocyte, and diamine oxidase (DAO), an enzyme that catalyzes oxidative deamination of histamine-like diamines, could reflect the degree of intestinal mucosal injury [39]. Huang et al. [25] reported that Huoxiang Zhengqi Oral Liquid reduced the DAO activity in the serum of rats with dampness obstructing spleen-stomach syndrome. In the present study, we found that the levels of serum iFABP and DAO increased after HS, but these increases were blunted in HZDP rats. Huoxiang Zhengqi Oral Liquid could also repair the intestinal mechanical barrier function by raising the expression levels of occludin and ZO-1 in rat colon tissues [25]. Similar results were observed, and HZDP restored the intestinal barrier by increasing the expression of claudin-3. Although claudins are the key proteins of TJs, it is unclear whether the interactions of claudin and occludin, ZO proteins, and claudin monomers participate in the regulation of TJs during heat exposure, as well as the mechanisms of these regulations.

It has been found that some intestinal cytokines, including IL-1*β*, IL-2, IL-6, IL-10, IL-12, and TNF-*α*, showed significant changes at different time points after heat stress in mice [31]. Considering the developmental process of intestinal injury and the inflammatory response during HS, the levels of serum inflammatory factors and intestinal-related proteins at different time points should be detected not only 2 h after HS. Since the expression of claudins varies in different intestinal segments [40], the tissue of other intestinal sites should also be analyzed, not only the jejunum. We are willing to observe more time points and more intestinal sites in future studies.

The intestine seems to be more susceptible to heat injury than other visceral organs, and direct intestinal injury caused by heat stress plays a key role in the pathogenesis and pathophysiology of HS [41–43]. Studies have shown that many components had the effects of improving intestinal injury and heat resistance. Wogonin, an ingredient of Atractylodis Rhizoma, has been shown to suppress the inflammatory response and maintain intestinal barrier function [44]. Hesperidin, the active ingredient of Citri Reticulatae Pericarpium, could protect against intestinal inflammation by reducing the levels of TNF-*α*, IL-6, and other inflammatory factors and increasing the levels of anti-inflammatory factors. Additionally, hesperidin could maintain intestinal barrier function by improving the expression of tight junction proteins and intestinal permeability in DSS-induced mouse colonic tissues [45, 46]. Magnolol and honokiol are the main bioactive compounds isolated from Magnoliae Officinalis Cortex. Previous studies demonstrated that magnolol and honokiol could enhance the intestinal anti-inflammatory capacities, elongate the villus height and crypt depth, and reduce goblet cell numbers to protect the intestinal mucosa [47]. Magnolol treatment attenuated dextran sulfate sodium-induced colitis by regulating inflammation and intestinal barrier integrity in mice [48]. Bioinformatics screening and the network pharmacology results revealed that TNF and IL-6 were the main inflammatory target genes related to HZDP. Hesperidin, magnolol, and other active components might have anti-inflammatory effect through inhibiting NF-*κ*B-mediated inflammatory cytokines mRNA formation [49], downregulation of p38/MAPK [50], blocking RIPK3/MLKL necroptosis signaling [45], and modulation of the JAk2/STAT3/SOCS3 pathway [51]. We speculated that HZDP might prevent intestinal injury mainly by wogonin, hesperidin, magnolol, magnolol, and other active components through anti-inflammatory effects, increasing the expression of tight junction proteins, maintaining villus morphology, and eventually improving heat resistance.

## 5. Conclusion

Our study demonstrated that Huoxiang Zhengqi Dropping Pills (HZDP) could effectively protect intestinal barrier function and prevent acute intestinal injury, eventually improving heat resistance. This study may provide a basis for minimizing the incidence of heatstroke by preventing and mitigating intestinal injury. It is worth considering that HZDP may serve as a valuable preventive medicine for heatstroke.

## Figures and Tables

**Table 1:** The 93 potential target genes of Huoxiang Zhengqi Dropping Pills prevented heatstroke.

No.	Target	Symbol	Entry	No.	Target	Symbol	Entry
1	Thrombin	F2	P00734	48	Progesterone receptor	PGR	P06401
2	Nitric oxide synthase, endothelial	NOS3	P29474	49	Alpha-1A adrenergic receptor	ADRA1A	P35348
3	Sodium channel protein type 5 subunit alpha	SCN5A	Q14524	50	Calcium-activated potassium channel subunit alpha-1	KCNMA1	Q12791
4	Interleukin-6	IL-6	P05231	51	Transcription factor p65	RELA	Q04206
5	Matrix metalloproteinase-9	MMP9	P14780	52	Dipeptidyl peptidase IV	DPP4	P27487
6	Cellular tumor antigen p53	TP53	P04637	53	Leukotriene A-4 hydrolase	LTA4H	P09960
7	RAC-alpha serine/threonine-protein kinase	AKT1	P31749	54	Phosphatidylinositol-4,5-bisphosphate 3-kinase catalytic subunit, gamma isoform	PIK3CG	P48736
8	Caspase-3	CASP3	P42574	55	Alpha-1B adrenergic receptor	ADRA1B	P35368
9	Tumor necrosis factor	TNF	P01375	56	Acetylcholinesterase	ACHE	P22303
10	Estrogen receptor	ESR1	P03372	57	Alpha-2A adrenergic receptor	ADRA2A	P08913
11	Peroxisome proliferator-activated receptor gamma	PPARG	P37231	58	Potassium voltage-gated channel subfamily H member 2	KCNH2	Q12809
12	Coagulation factor VII	F7	P08709	59	Insulin-like growth factor II	IGF2	P01344
13	Beta-2 adrenergic receptor	ADRB2	P07550	60	Serine/threonine-protein kinase Chk1	CHEK1	O14757
14	Interleukin-8	CXCL8	P10145	61	Egl nine homolog 1	EGLN1	Q9GZT9
15	Serum paraoxonase/arylesterase 1	PON1	P27169	62	Sodium-dependent dopamine transporter	SLC6A3	Q01959
16	Mitogen-activated protein kinase 14	MAPK14	Q16539	63	Urokinase-type plasminogen activator	PLAU	P00749
17	Mitogen-activated protein kinase 8	MAPK8	P45983	64	Sodium-dependent noradrenaline transporter	SLC6A2	P23975
18	Coagulation factor Xa	F10	P00742	65	Aryl hydrocarbon receptor	AHR	P35869
19	Caspase-9	CASP9	P55211	66	Alpha-1D adrenergic receptor	ADRA1D	P25100
20	Prostaglandin G/H synthase 2	PTGS2	P35354	67	5-Hydroxytryptamine receptor 3A	HTR3A	P46098
21	Cyclic AMP-responsive element-binding protein 1	CREB1	P16220	68	Scavenger receptor cysteine-rich type 1 protein M130	CD163	Q86VB7
22	C-C motif chemokine 2	CCL2	P13500	69	Amine oxidase [flavin-containing] B	MAOB	P27338
23	Transforming growth factor beta-1	TGFB1	P01137	70	Neuronal acetylcholine receptor protein, alpha-7 chain	CHRNA7	P36544
24	Sodium-dependent serotonin transporter	SLC6A4	P31645	71	5-Hydroxytryptamine 2C receptor	HTR2C	P28335
25	Nitric oxide synthase, inducible	NOS2	P35228	72	Amine oxidase [flavin-containing] A	MAOA	P21397
26	Hypoxia-inducible factor 1-alpha	HIF1A	Q16665	73	Alpha-2C adrenergic receptor	ADRA2C	P18825
27	Caspase-8	CASP8	Q14790	74	Protein kinase C delta type	PRKCD	Q05655
28	Interstitial collagenase	MMP1	P03956	75	Cyclin-A2	CCNA2	P20248
29	Myeloperoxidase	MPO	P05164	76	Cytosolic phospholipase A2	PLA2G4A	P47712
30	Microtubule-associated protein 2	MAP2	P11137	77	Cell division protein kinase 2	CDK2	P24941
31	Androgen receptor	AR	P10275	78	NADPH oxidase 5	NOX5	Q96PH1
32	G1/S-specific cyclin-D1	CCND1	P24385	79	G2/mitotic-specific cyclin-B1	CCNB1	P14635
33	Apoptosis regulator Bcl-2	BCL2	P10415	80	Glutamate receptor 2	GRIA2	P42262
34	CGMP-inhibited 3',5'-cyclic phosphodiesterase A	PDE3A	Q14432	81	mRNA of protein-tyrosine phosphatase, nonreceptor type 1	PTPN1	P18031
35	Beta-1 adrenergic receptor	ADRB1	P08588	82	Apolipoprotein D	APOD	P05090
36	Metalloproteinase inhibitor 1	TIMP1	P01033	83	Retinoic acid receptor RXR-alpha	RXRA	P19793
37	Transcription factor AP-1	JUN	P05412	84	Fatty acid-binding protein 5	FABP5	Q01469
38	Prostaglandin G/H synthase 1	PTGS1	P23219	85	Muscarinic acetylcholine receptor M3	CHRM3	P20309
39	Proto-oncogene c-Fos	FOS	P01100	86	Prostaglandin E2 receptor EP3 subtype	PTGER3	P43115
40	Fibronectin	FN1	P02751	87	Telomerase protein component 1	TEP1	Q99973
41	Mineralocorticoid receptor	NR3C2	P08235	88	Carbonic anhydrase II	CA2	P00918
42	Apoptosis regulator BAX	BAX	Q07812	89	Muscarinic acetylcholine receptor M2	CHRM2	P08172
43	Glycogen synthase kinase-3 beta	GSK3B	P49841	90	Delta-type opioid receptor	OPRD1	P41143
44	5-Hydroxytryptamine 2A receptor	HTR2A	P28223	91	Lysozyme	LYZ	P61626
45	Mu-type opioid receptor	OPRM1	P35372	92	Ephrin type-B receptor 2	EPHB2	P29323
46	Purine nucleoside phosphorylase	PNP	P00491	93	Bcl-2-binding component 3	BBC3	Q96PG8
47	Protein kinase C alpha type	PRKCA	P17252				

## Data Availability

The data used to support the findings of this study are available from the corresponding author upon request.

## Abbreviations

HZDP: Huoxiang Zhengqi Dropping Pills
HS: Heatstroke
TNF-*α*: Tumor necrosis factor-*α*
IL-6: Interleukin-6
iFABP: Intestinal fatty acid-binding protein
DAO: Diamine oxidase.

## Supplementary Materials

Supplementary Materials
*Table S1.* Characteristics of active ingredients in Huoxiang Zhengqi Dropping Pills. *Table S2*. The 128 target genes of Huoxiang Zhengqi Dropping Pills active ingredients.
