# Supplementary material for: The Chinese Herbal Formula Huoxiang Zhengqi Dropping Pills Prevents Acute Intestinal Injury Induced by Heatstroke by Increasing the Expression of Claudin-3 in Rats
Source: Evid Based Complement Alternat Med. 2022 Jul 31;2022:9230341. doi: 10.1155/2022/9230341 (PMC9357687; doi:10.1155/2022/9230341)
Supplement: Supplementary Materials — Table S1. Characteristics of active ingredients in Huoxiang Zhengqi Dropping Pills. Table S2. The 128 target genes of Huoxiang Zhengqi Dropping Pills active ingredients. [file 9230341.f1.docx]

***Supplementary Material***

**Table S1.** Characteristics of active ingredients in Huoxiang Zhengqi Dropping Pills

| No | Molecule ID | Molecule name | Molecular weight | OB (%) | DL |
| --- | --- | --- | --- | --- | --- |
| 1 | MOL000173 | wogonin | 284.28 | 30.68 | 0.23 |
| 2 | MOL000188 | 3β-acetoxyatractylone | 274.39 | 40.57 | 0.22 |
| 3 | MOL000359 | sitosterol | 414.79 | 36.91 | 0.75 |
| 4 | MOL005828 | nobiletin | 402.43 | 61.67 | 0.52 |
| 5 | MOL005815 | Citromitin | 404.45 | 86.90 | 0.51 |
| 6 | MOL005100 | 5,7-dihydroxy-2-(3-hydroxy-4-methoxyphenyl)chroman-4-one | 302.30 | 47.74 | 0.27 |
| 7 | MOL004328 | naringenin | 272.27 | 59.29 | 0.21 |
| 8 | MOL005970 | Eucalyptol | 266.36 | 60.62 | 0.32 |
| 9 | MOL005980 | Neohesperidin | 302.30 | 57.44 | 0.27 |
| 10 | MOL005972 | OBOVATOL | 282.36 | 69.45 | 0.18 |
| 11 | MOL000449 | Stigmasterol | 412.77 | 43.83 | 0.76 |
| 12 | MOL000358 | beta-sitosterol | 414.79 | 36.91 | 0.75 |
| 13 | MOL005807 | sen-byakangelicol | 386.43 | 58.00 | 0.61 |
| 14 | MOL005789 | neobyakangelico l | 316.33 | 36.18 | 0.31 |
| 15 | MOL001956 | Cnidilin | 300.33 | 32.69 | 0.28 |
| 16 | MOL002644 | Phellopterin | 300.33 | 40.19 | 0.28 |
| 17 | MOL005792 | {5-[2'(R)-Hydroxy-3'-methyl-3'-butenyl-oxy]furocoumarin} | 286.30 | 42.85 | 0.26 |
| 18 | MOL001941 | Ammidin | 270.30 | 34.55 | 0.22 |
| 19 | MOL003588 | Prangenidin | 270.30 | 36.31 | 0.22 |
| 20 | MOL001939 | Alloisoimperatorin | 270.30 | 34.80 | 0.22 |
| 21 | MOL004793 | Marmesine | 246.28 | 84.77 | 0.18 |
| 22 | MOL000283 | Ergosterol peroxide | 430.74 | 40.36 | 0.81 |
| 23 | MOL000296 | hederagenin | 414.79 | 36.91 | 0.75 |
| 24 | MOL000004 | Procyanidin B1 | 578.56 | 67.87 | 0.66 |
| 25 | MOL000073 | ent-Epicatechin | 290.29 | 48.96 | 0.24 |
| 26 | MOL002670 | Cavidine | 353.45 | 35.64 | 0.81 |
| 27 | MOL003578 | Cycloartenol | 426.80 | 38.69 | 0.78 |
| 28 | MOL001755 | 24-Ethylcholest-4-en-3-one | 412.77 | 36.08 | 0.76 |
| 29 | MOL000449 | Stigmasterol | 412.77 | 43.83 | 0.76 |
| 30 | MOL002776 | Baicalin | 446.39 | 40.12 | 0.75 |
| 31 | MOL000358 | beta-sitosterol | 414.79 | 36.91 | 0.75 |
| 32 | MOL000519 | coniferin | 314.41 | 31.11 | 0.32 |
| 33 | MOL006957 | (3S,6S)-3-(benzyl)-6-(4-hydroxybenzyl)piperazine-2,5-quinone | 310.38 | 46.89 | 0.27 |
| 34 | MOL002714 | baicalein | 270.25 | 33.52 | 0.21 |
| 35 | MOL006967 | beta-D-Ribofuranoside, xanthine-9 | 284.26 | 44.72 | 0.21 |

**Table S2.** The 128 target genes of Huoxiang Zhengqi Dropping Pills active ingredients

| No. | Target | Symbol | Entry | No. | Target | Symbol | Entry |
| --- | --- | --- | --- | --- | --- | --- | --- |
| 1 | Thrombin | F2 | P00734 | 65 | Aryl hydrocarbon receptor | AHR | P35869 |
| 2 | Nitric-oxide synthase , endothelial | NOS3 | P29474 | 66 | Alpha-1D adrenergic receptor | ADRA1D | P25100 |
| 3 | Sodium channel protein type 5 subunit alpha | SCN5A | Q14524 | 67 | 5-hydroxytryptamine receptor 3A | HTR3A | P46098 |
| 4 | Interleukin-6 | IL6 | P05231 | 68 | Scavenger receptor cysteine-rich type 1 protein M130 | CD163 | Q86VB7 |
| 5 | Matrix metalloproteinase-9 | MMP9 | P14780 | 69 | Amine oxidase [flavin-containing] B | MAOB | P27338 |
| 6 | Cellular tumor antigen p53 | TP53 | P04637 | 70 | Neuronal acetylcholine receptor protein, alpha-7 chain | CHRNA7 | P36544 |
| 7 | RAC-alpha serine/ threonine- protein kinase | AKT1 | P31749 | 71 | 5-hydroxytryptamine 2C receptor | HTR2C | P28335 |
| 8 | Caspase-3 | CASP3 | P42574 | 72 | Amine oxidase [flavin-containing] A | MAOA | P21397 |
| 9 | Tumor necrosis factor | TNF | P01375 | 73 | Alpha-2C adrenergic receptor | ADRA2C | P18825 |
| 10 | Estrogen receptor | ESR1 | P03372 | 74 | Protein kinase C delta type | PRKCD | Q05655 |
| 11 | Peroxisome proliferator activated receptor gamma | PPARG | P37231 | 75 | Cyclin-A2 | CCNA2 | P20248 |
| 12 | Coagulation factor VII | F7 | P08709 | 76 | Cytosolic phospholipase A2 | PLA2G4A | P47712 |
| 13 | Beta-2 adrenergic receptor | ADRB2 | P07550 | 77 | Cell division protein kinase 2 | CDK2 | P24941 |
| 14 | Interleukin-8 | CXCL8 | P10145 | 78 | NADPH oxidase 5 | NOX5 | Q96PH1 |
| 15 | Serum paraoxonase/arylesterase 1 | PON1 | P27169 | 79 | G2/mitotic-specific cyclin-B1 | CCNB1 | P14635 |
| 16 | Mitogen-activated protein kinase 14 | MAPK14 | Q16539 | 80 | Glutamate receptor 2 | GRIA2 | P42262 |
| 17 | Mitogen-activated protein kinase 8 | MAPK8 | P45983 | 81 | mRNA of Protein-tyrosine phosphatase, non-receptor type 1 | PTPN1 | P18031 |
| 18 | Coagulation factor Xa | F10 | P00742 | 82 | Apolipoprotein D | APOD | P05090 |
| 19 | Caspase-9 | CASP9 | P55211 | 83 | Retinoic acid receptor RXR-alpha | RXRA | P19793 |
| 20 | Prostaglandin G/H synthase 2 | PTGS2 | P35354 | 84 | Fatty acid-binding protein 5 | FABP5 | Q01469 |
| 21 | Cyclic AMP-responsive element-binding protein 1 | CREB1 | P16220 | 85 | Muscarinic acetylcholine receptor M3 | CHRM3 | P20309 |
| 22 | C-C motif chemokine 2 | CCL2 | P13500 | 86 | Prostaglandin E2 receptor EP3 subtype | PTGER3 | P43115 |
| 23 | Transforming growth factor beta-1 | TGFB1 | P01137 | 87 | Telomerase protein component 1 | TEP1 | Q99973 |
| 24 | Sodium-dependent serotonin transporter | SLC6A4 | P31645 | 88 | Carbonic anhydrase II | CA2 | P00918 |
| 25 | Nitric oxide synthase , inducible | NOS2 | P35228 | 89 | Muscarinic acetylcholine receptor M2 | CHRM2 | P08172 |
| 26 | Hypoxia-inducible factor 1-alpha | HIF1A | Q16665 | 90 | Delta-type opioid receptor | OPRD1 | P41143 |
| 27 | Caspase-8 | CASP8 | Q14790 | 91 | Lysozyme | LYZ | P61626 |
| 28 | Interstitial collagenase | MMP1 | P03956 | 92 | Ephrin type-B receptor 2 | EPHB2 | P29323 |
| 29 | Myeloperoxidase | MPO | P05164 | 93 | Bcl-2-binding component 3 | BBC3 | Q96PG8 |
| 30 | Microtubule-associated protein 2 | MAP2 | P11137 | 94 | Nuclear receptor coactivator 2 | NCOA2 | Q15596 |
| 31 | Androgen receptor | AR | P10275 | 95 | Muscarinic acetylcholine receptor M1 | CHRM1 | P11229 |
| 32 | Calcium-activated potassium channel subunit alpha 1 | KCNMA1 | Q12791 | 96 | Gamma-aminobutyric-acid receptor alpha-2 subunit | GABRA2 | P47869 |
| 33 | Potassium voltage-gated channel subfamily H member 2 | KCNH2 | Q12809 | 97 | Gamma-aminobutyric-acid receptor alpha-3 subunit | GABRA3 | P34903 |
| 34 | CGMP-inhibited 3',5'-cyclic phosphodiesterase A | PDE3A | Q14432 | 98 | Gamma-aminobutyric acid receptor subunit alpha-1 | GABRA1 | P14867 |
| 35 | Phosphatidylinositol-4,5-bisphosphate 3-kinase catalytic subunit, gamma isoform | PIK3CG | P48736 | 99 | Gamma-aminobutyric-acid receptor subunit alpha-6 | GABRA6 | Q16445 |
| 36 | Sodium-dependent noradrenaline transporter | SLC6A2 | P23975 | 100 | Gamma-aminobutyric-acid receptor alpha-5 subunit | GABRA5 | P31644 |
| 37 | Transcription factor AP-1 | JUN | P05412 | 101 | Alcohol dehydrogenase 1B | ADH1B | A0A024RDE3 |
| 38 | Prostaglandin G/H synthase 1 | PTGS1 | P23219 | 102 | Alcohol dehydrogenase 1C | ADH1C | P00326 |
| 39 | Proto-oncogene c-Fos | FOS | P01100 | 103 | Heat shock protein HSP 90-beta | HSP90AB1 | P08238 |
| 40 | Fibronectin | FN1 | P02751 | 104 | Heat shock protein HSP 90-alpha | HSP90AA1 | P07900 |
| 41 | Mineralocorticoid receptor | NR3C2 | P08235 | 105 | cAMP-dependent protein kinase inhibitor alpha | PKIA | P61925 |
| 42 | Apoptosis regulator BAX | BAX | Q07812 | 106 | Nuclear receptor coactivator 1 | NCOA1 | Q15788 |
| 43 | Glycogen synthase kinase-3 beta | GSK3B | P49841 | 107 | Chymotrypsinogen B | CTRB1 | P17538 |
| 44 | 5-hydroxytryptamine 2A receptor | HTR2A | P28223 | 108 | Muscarinic acetylcholine receptor M4 | CHRM4 | P08173 |
| 45 | Mu-type opioid receptor | OPRM1 | P35372 | 109 | Neuronal acetylcholine receptor subunit alpha-2 | CHRNA2 | Q15822 |
| 46 | cAMP and cAMP-inhibited cGMP 3',5'-cyclic phosphodiesterase 10A | PDE10A | Q9Y233 | 110 | Proto-oncogene serine/threonine-protein kinase Pim-1 | PIM1 | P11309 |
| 47 | Protein kinase C alpha type | PRKCA | P17252 | 111 | DNA topoisomerase II | TOP2 | E9PCY5 |
| 48 | Progesterone receptor | PGR | P06401 | 112 | Trypsin-1 | PRSS1 | P07477 |
| 49 | Alpha-1A adrenergic receptor | ADRA1A | P35348 | 113 | Calmodulin-1 | CALM1 | P0DP23 |
| 50 | G1/S-specific cyclin-D1 | CCND1 | P24385 | 114 | Calmodulin-2 | CALM2 | P0DP24 |
| 51 | Transcription factor p65 | RELA | Q04206 | 115 | Calmodulin-3 | CALM3 | P0DP25 |
| 52 | Dipeptidyl peptidase IV | DPP4 | P27487 | 116 | Muscarinic acetylcholine receptor M5 | CHRM5 | P08912 |
| 53 | Leukotriene A-4 hydrolase | LTA4H | P09960 | 117 | Cyclin-dependent kinase inhibitor 1 | CDKN1A | P38936 |
| 54 | Beta-1 adrenergic receptor | ADRB1 | P08588 | 118 | Eukaryotic translation initiation factor 6 | EIF6 | P56537 |
| 55 | Alpha-1B adrenergic receptor | ADRA1B | P35368 | 119 | Vascular endothelial growth factor receptor 2 | KDR | P35968 |
| 56 | Induced myeloid leukemia cell differentiation protein Mcl-1 | MCL1 | Q07820 | 120 | Activator of 90 kDa heat shock protein ATPase homolog 1 | AHSA1 | O95433 |
| 57 | Alpha-2A adrenergic receptor | ADRA2A | P08913 | 121 | Acetylcholinesterase | ACHE | P22303 |
| 58 | Apoptosis regulator Bcl-2 | BCL2 | P10415 | 122 | Retinoic acid receptor RXR-beta | RXRB | P28702 |
| 59 | Insulin-like growth factor II | IGF2 | P01344 | 123 | Purine nucleoside phosphorylase | PNP | P00491 |
| 60 | Serine/threonine-protein kinase Chk1 | CHEK1 | O14757 | 124 | Fos-related antigen 1 | FOSL1 | P15407 |
| 61 | Egl nine homolog 1 | EGLN1 | Q9GZT9 | 125 | Fos-related antigen 2 | FOSL2 | P15408 |
| 62 | Sodium-dependent dopamine transporter | SLC6A3 | Q01959 | 126 | Cytochrome c | CYCS | P99999 |
| 63 | Urokinase-type plasminogen activator | PLAU | P00749 | 127 | Nuclear factor of activated T-cells, cytoplasmic 1 | NFATC1 | O95644 |
| 64 | Metalloproteinase inhibitor 1 | TIMP1 | P01033 | 128 | Tudor domain-containing protein 7 | TDRD7 | Q8NHU6 |
